# A Rare Case of Epstein-Barr Virus Hepatitis and Rash in an Adolescent

**DOI:** 10.7759/cureus.85353

**Published:** 2025-06-04

**Authors:** Brendan Coyne, Mariam B Elghazzawy

**Affiliations:** 1 Department of Psychiatry, George Washington University School of Medicine and Health Sciences, Washington, DC, USA

**Keywords:** acute viral hepatitis, case report in pediatrics, cholestatic liver injury, epstein-barr virus, human herpes virus, infectious mononucleosis syndrome, maculopapular rash, reactive lymphadenitis, ursodiol, young female with abdominal pain

## Abstract

Infection from Epstein-Barr virus (EBV) manifests with a diverse spectrum of presentations. One common manifestation is hepatobiliary involvement with hepatitis, which in most cases is mild and self-limited. Severe or fulminant hepatitis can potentially occur, though this is rare, particularly in immunocompetent pediatric populations. We present a previously healthy female adolescent with severe abdominal pain and jaundice, but no lymph node or tonsillar swelling. The workup revealed a marked elevation in liver enzymes, bilirubin, and mesenteric adenitis. A positive polymerase chain reaction (PCR) for EBV confirmed the diagnosis of EBV hepatitis and cholestasis. The patient was primarily managed supportively, including intravenous (IV) hydration. During her treatment course, she developed a diffuse maculopapular rash of uncertain etiology, which gradually self-resolved. We review similar cases reported in the literature, as well as potentially life-threatening complications of EBV infection. It is therefore prudent to consider EBV hepatitis in patients with abdominal pain and transaminitis, even in the absence of immunocompromise or classic mononucleosis symptoms.

## Introduction

Epstein-Barr virus (EBV) is a human herpesvirus known to cause the majority of infectious mononucleosis cases [1]. Particularly among young children, EBV infection is usually mild or asymptomatic; however, older children and adolescents carry a higher risk of developing full-blown mononucleosis, with symptoms such as fever, lymphadenopathy, and hepatosplenomegaly [2,3]. 

One of the common hepatobiliary manifestations of EBV infection is transaminitis, particularly in adults; however, this is often subclinical and self-limiting [4,5]. Rarely, EBV may cause acute acalculous cholecystitis [6,7] or hepatitis [8]. Marked elevation in liver enzymes accompanied by cholestasis often indicates a complicated mononucleosis course or a co-infection from hepatitis viruses [9,10].

Diagnosis of EBV hepatitis is usually based on a combination of clinical and laboratory findings, which include lymphocytosis with numerous atypical lymphocytes, elevated liver function tests (LFTs), and positive serology for EBV and/or heterophile antibodies [11,12]. It is quite rare for EBV hepatitis to progress into severe, life-threatening liver failure in young immunocompetent individuals, but it is nonetheless possible [8].

## Case presentation

A 16-year-old female patient with no significant past medical history presented with a chief complaint of diffuse abdominal pain. These symptoms had initially begun three days prior and were accompanied by generalized fatigue, fever, and yellow skin discoloration. On admission, her vital signs demonstrated a fever of up to 40 °C, tachycardia at 127 beats per minute, stable blood pressure, and a normal respiratory rate and pulse oximetry on room air.

On examination, the patient was overall well-appearing. Her skin was mildly jaundiced, but there was no scleral icterus. Her abdominal exam showed mild distention and marked tenderness to palpation of the bilateral upper quadrants with voluntary guarding and positive Murphy’s sign. However, there was no rigidity, masses, or hepatosplenomegaly. Tonsils appeared normal, there was no palpable cervical lymphadenopathy, and the remainder of the exam was unremarkable. 

Lab workup revealed no significant electrolyte abnormalities but was positive for conjugated hyperbilirubinemia, transaminitis, and elevated alkaline phosphatase (ALP) and gamma-glutamyl transferase (GGT) (Table 1). The patient did not have leukocytosis, though she did have bandemia. The overall findings raised concern for a possible hepatobiliary process such as acute acalculous cholecystitis or ascending cholangitis. This prompted broad-spectrum antibiotic coverage with piperacillin-tazobactam.

**Table 1:** Patient labs on admission AST: aspartate aminotransferase, ALT: alanine aminotransferase, ALP: alkaline phosphatase, GGT: gamma-glutamyl transferase.

Laboratory test	Result	Reference range
Sodium	137 mEq/L	135-145 mEq/L
Potassium	3.6 mEq/L	3.5-5.0 mEq/L
Chloride	105 mEq/L	98-107 mEq/L
Calcium	8.0 mg/dL	8.5-10.5 mg/dL
Total bilirubin	3.3 mg/dL	0.1-1.2 mg/dL
Direct bilirubin	2.9 mg/dL	0.0-0.3 mg/dL
AST	480 U/L	10-40 U/L
ALT	384 U/L	7-56 U/L
ALP	145 U/L	44-147 U/L
GGT	136 U/L	0-26 U/L
Ferritin	373 μg/L	24-307 μg/L
Albumin	2.7 g/dL	3.5-5.0 g/dL

**Figure 1:**
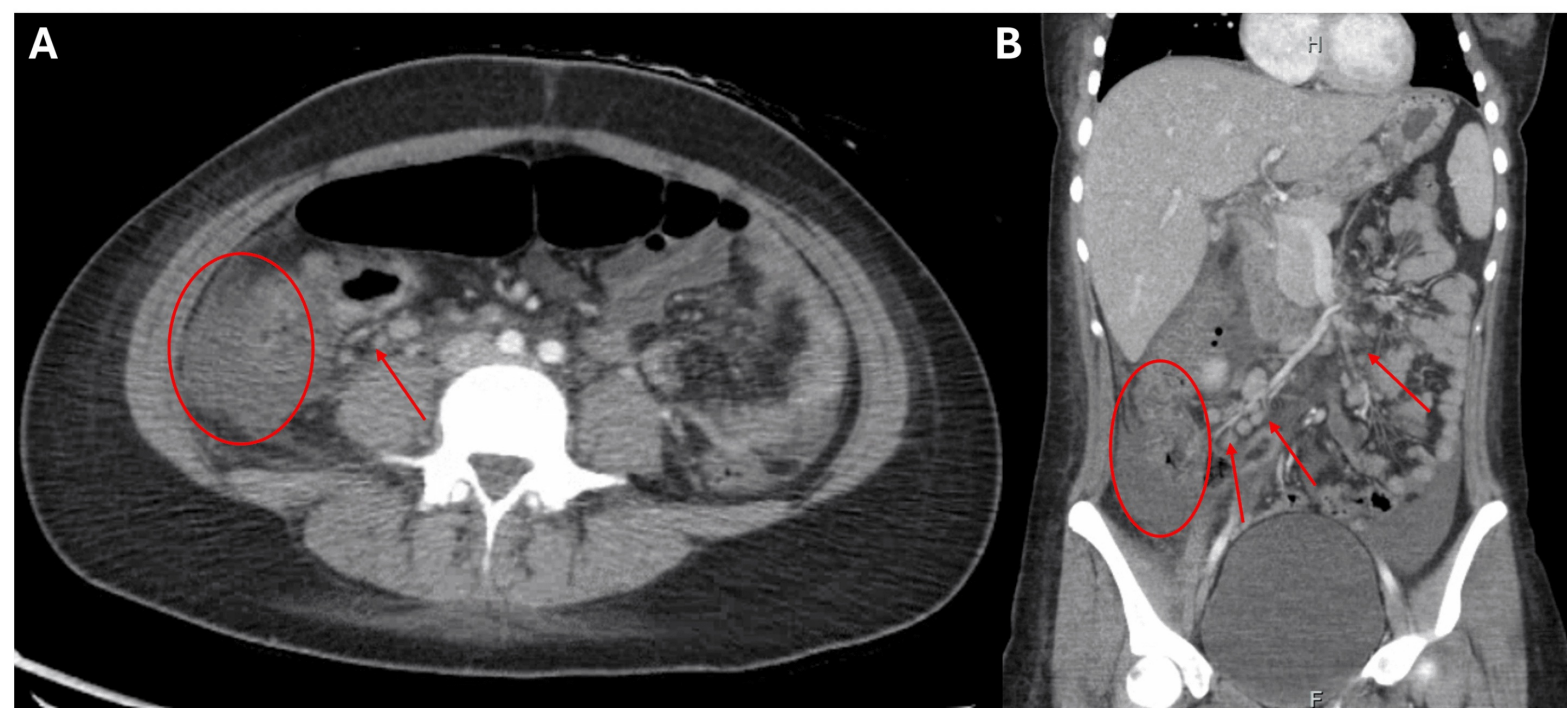
Patient’s CT imaging of the abdomen and pelvis with contrast (A) Transverse section of the patient’s abdomen. (B) Coronal section of the patient’s abdomen. There was nonspecific right-sided colitis with ascending colon wall thickening, hyperemia, and adjacent fat stranding (ovals). There was also diffuse mesenteric adenitis with mildly enlarged lymph nodes, most prominently in the right lower quadrant (arrows).

Ultrasound (US) and computed tomography (CT) of the abdomen revealed a non-distended gallbladder with mild diffuse thickening of the wall but with no pericholecystic fluid. There was a moderate amount of ascites, but the liver parenchyma was normal in appearance, and there were no signs of calcified cholelithiasis or biliary tree dilatation. There was nonspecific right-sided (cecum to hepatic flexure) colitis with adjacent mesenteric adenitis and inflammatory changes (Figure 1).

A thorough infectious workup revealed a positive quantitative polymerase chain reaction (PCR) for EBV, with 55,624 copies/mL detected. PCRs for cytomegalovirus (CMV), influenza A and B, and severe acute respiratory syndrome coronavirus 2 (SARS-CoV-2) were negative. Serologies for hepatitis viruses A, B, C, D, and E were negative. With multiple negative bacterial cultures and stable white blood cell (WBC) levels, the patient’s piperacillin-tazobactam was discontinued after four days of treatment. Supportive management was continued with intravenous (IV) hydration and 400 mg of ibuprofen as needed for fevers and pain. Additionally, 300 mg of ursodiol was started. Acetaminophen was avoided due to concerns of liver injury.

On day 9 of admission, the patient developed a new-onset maculopapular rash with pruritus and dark-colored urine in the setting of rising LFTs and elevated urine urobilinogen of 4.0 mg/dL (Table 2). Skin exam showed diffuse, erythematous, blanching rash with areas of confluence over the bilateral upper and lower extremities and torso with sparing of the palms and soles. A repeat abdominal US was reassuring with no acute changes.

**Table 2:** Patient's hepatobiliary lab trends AST: aspartate aminotransferase, ALT: alanine aminotransferase, ALP: alkaline phosphatase, GGT: gamma-glutamyl transferase.

Laboratory test (reference range)	Day 1 (admission)	Day 3	Day 8	Day 10 (discharge)	Units
Total bilirubin (0.1-1.2)	3.3	4.2	6.9	5.8	mg/dL
Direct bilirubin (0.0-0.3)	2.9	3.7	6.0	5.2	mg/dL
AST (10-40)	480	345	162	196	U/L
ALT (7-56)	384	366	155	159	U/L
ALP (44-147)	145	171	454	532	U/L
GGT (0-26)	136	---	149	205	U/L

The skin findings were suspected to be a mononucleosis rash, but ursodiol was discontinued due to the theoretical possibility of a hypersensitivity reaction. The patient was given one dose of oral cetirizine before switching to oral hydroxyzine twice a day as needed, which was continued outpatient. The patient’s pruritic symptoms rapidly improved following these measures, although the rash itself did not immediately resolve.

The patient’s urine discoloration self-resolved and there were no further episodes. This was thought to be secondary to the cholestasis and urobilinogenuria. With the patient stabilized and with no other complications or concerns, she was safely discharged with recommendations to avoid strenuous or contact sports for one month. Outpatient follow-up revealed continued improvement of symptoms, with a resolution of her rash within two weeks without recurrence.

## Discussion

The pathophysiology of EBV hepatitis is not definitively known but may be secondary to a combination of systemic inflammation and direct invasion into the liver parenchyma by EBV-infected B cells [13]. The obstructive mechanism for EBV-related cholestasis is thought to be related to bile duct swelling as opposed to viral infection of the duct’s epithelial cells [14,15]. EBV-related cholestatic hepatitis and mesenteric lymphadenitis are most often reported in adult cases [16-23]. Data in the pediatric population is limited, but one study did find that among children hospitalized with EBV infection, transient cholestatic liver disease was the predominant form of hepatic involvement [13]. The majority of these patients were older than 15 years (66.6%), some with darkened urine (44.4%), and all of whom presented with jaundice, abdominal tenderness, and pruritus [13]. Another study of the pediatric population found that EBV hepatitis can present with jaundice and lymphadenitis even in the absence of classic mononucleosis symptoms [24]. Our findings are consistent with these studies. Additionally, the fluctuating nature of our patient’s liver enzymes and hyperbilirubinemia is congruent with the disease course of EBV hepatitis. For example, according to Rutkowska and Pokorska-Śpiewak, LFTs were most elevated during the first three weeks from disease onset, with biphasic peaks [13]. 

EBV hepatitis is typically self-limiting, and standard treatment is primarily supportive [10,25]. Some authors have reported utilizing corticosteroids, antivirals, or alternative treatments (e.g., high-dose ascorbic acid) for severe cases [26,27]. However, this remains controversial due to limited data and a lack of robust clinical trials [28,29]. Thus, our patient was primarily managed with supportive measures. 

During her treatment course, our patient developed a diffuse rash. This has been reported in previous cases of EBV-induced cholestatic hepatitis, although it is an overall rare manifestation in the pediatric population [24,30]. The etiology of our patient’s rash was not precisely clear. Alpha-1 antitrypsin deficiency was ruled out given the character of the rash and the patient’s normal MM phenotype. Cholestatic pruritus was unlikely as it does not usually present with a primary rash in non-pregnant individuals. Ibuprofen or ursodiol hypersensitivity was possible but is rare and would be expected to develop immediately following first exposure/administration [31]. Certain antibiotics can induce rashes in EBV patients up to 10 days following initial exposure [32,33]. However, the incidence is overall low, and neither piperacillin nor tazobactam is the common cause [33]. Thus, it is believed that our patient’s symptoms potentially reflected a mononucleosis skin rash due to the virus itself.

Interestingly, our patient also presented with very mildly elevated ferritin levels of 373 μg/L. Ferritin is considered an acute-phase reactant; significantly elevated ferritin in the setting of EBV infection may indicate complications such as severe mononucleosis [34], autoimmune hemolytic anemia [35,36], or hemophagocytic lymphohistiocytosis (HLH) [37]. Given our patient’s elevated ferritin, LFTs, triglycerides, and persistent fever, there was an initial concern for HLH. However, ferritin levels in HLH surpass 500 μg/L and oftentimes 1000 μg/L [37]. HLH is exceedingly rare in young, immunocompetent individuals [30,38], and our patient did not meet the diagnostic criteria (Table 3) [39,40].

**Table 3:** Diagnostic criteria for HLH HLH: hemophagocytic lymphohistiocytosis.

Diagnosis of HLH requires:
(A) A molecular diagnosis consistent with HLH.
AND/OR
(B) Five of the following eight clinical and laboratory findings:
Fever > 38.5 °C
Splenomegaly on exam
Cytopenia affecting at least two lineages, with hemoglobin < 9 g/dL, platelets < 100 × 10^9^/L, and/or neutrophils < 1.0 × 10^9^/L
Hypertriglyceridemia (fasting triglycerides > 265 mg/dL) and/or hypofibrinogenemia (fibrinogen ≤ 1.5 g/L)
Pathological evidence of hemophagocytosis in the bone marrow, spleen, liver, lymph nodes, or other tissues
Low or absent natural killer cell activity
Serum ferritin concentration ≥ 500 μg/L
Soluble CD25 (an IL-2 receptor) ≥ 2400 U/mL

With no evidence of HLH or autoimmune hemolytic anemia, our patient’s mild ferritin elevation was considered to be isolated. Isolated hyperferritinemia in EBV hepatitis is a rare presentation, though it has been documented in adults in recent literature [41,42]. This is the first known report of isolated hyperferritinemia in an immunocompetent adolescent with EBV hepatitis.

## Conclusions

Our case is a demonstration of the numerous ways in which EBV infection may present. Severe hepatitis is a rare but important manifestation. It is therefore important to consider a diagnosis of EBV hepatitis in patients with abdominal pain, mesenteric adenitis, elevated LFTs, and/or hyperbilirubinemia, even in the absence of classic mononucleosis symptoms. Management is primarily supportive, and providers should ensure to rule out severe, potentially life-threatening complications, such as HLH.
